# Sec62 Regulates Endoplasmic Reticulum Stress and Autophagy Balance to Affect Foot-and-Mouth Disease Virus Replication

**DOI:** 10.3389/fcimb.2021.707107

**Published:** 2021-08-31

**Authors:** Jin’en Wu, Zhihui Zhang, Zhidong Teng, Sahibzada Waheed Abdullah, Shiqi Sun, Huichen Guo

**Affiliations:** ^1^State Key Laboratory of Veterinary Etiological Biology and National Foot-and-Mouth Disease Reference Laboratory, Lanzhou Veterinary Research Institute, Chinese Academy of Agricultural Sciences, Lanzhou, China; ^2^College of Animal Science, Yangtze University, Jingzhou, China

**Keywords:** FMDV, ER stress, autophagy, Sec62, UPR

## Abstract

Endoplasmic reticulum (ER) stress-induced autophagy is closely associated with viral infection and propagation. However, the intrinsic link between ER stress, autophagy, and viral replication during foot-and-mouth disease virus (FMDV) infection is not fully elucidated. Our previous studies demonstrated that FMDV infection activated the ER stress-associated UPR of the PERK-eIF2a and ATF6 signaling pathway, whereas the IRE1a signaling was suppressed. We found that the activated-ATF6 pathway participated in FMDV-induced autophagy and FMDV replication, while the IRE1α pathway only affected FMDV replication. Further studies indicated that Sec62 was greatly reduced in the later stages of FMDV infection and blocked the activation of the autophagy-related IRE1α-JNK pathway. Moreover, it was also found that Sec62 promoted IRE1a phosphorylation and negatively regulated FMDV proliferation. Importantly, Sec62 may interact with LC3 to regulate ER stress and autophagy balance and eventually contribute to FMDV clearance *via* fusing with lysosomes. Altogether, these results suggest that Sec62 is a critical molecule in maintaining and recovering ER homeostasis by activating the IRE1α-JNK pathway and delivering autophagosome into the lysosome, thus providing new insights on FMDV-host interactions and novel antiviral therapies.

## Introduction

Foot-and-mouth disease virus (FMDV), the member of the *Aphthovirus* genus within the *Picornaviridae* family, contains seven serotypes (A, C, O, Asia, SAT1, SAT2, and SAT3) and is the causative agent of foot-and-mouth disease (FMD), which is the first legal infectious disease listed by the World Organization for Animal Health (Klein, 2009). FMDV is the most economically important and highly contagious veterinary pathogen, affecting long-term livestock health and international trade (Grubman and Baxt, 2004; Dong et al., 2021). Although the outbreaks of FMD have been effectively controlled by various prophylactic vaccines and animal culling (Jamal and Belsham, 2013; Abdullah et al., 2020), the potential for future outbreaks warns us of the need for safe and effective therapeutics to prevent viral infections. Growing researches have demonstrated that many viruses can induce endoplasmic reticulum (ER) stress-mediated autophagy to regulate viral replication and pathogenesis (Lv et al., 2015; Sharma et al., 2017; Lee et al., 2018). However, the detailed mechanisms involved in the rapid replication of FMDV, infection, and pathogenesis still need further investigations.

ER stress is a general term for various stimuli (hypoxia, Ca^+^ imbalance, starvation, and viral infections), leading to the accumulation of misfolded and unfolded proteins and then affecting ER functions (Kaufman, 1999). Upon ER stress, a series of complementary adaptive mechanisms are activated, including the unfolded protein response (UPR) and ER-associated degradation (ERAD), to reduce a load of newly synthesized proteins within the ER lumen and eliminate inappropriately folded proteins for cell function and survival (Hampton, 2000; Yoshida et al., 2003). The UPR is a highly conserved mechanism that enables to reduce of ER stress and recovery ER homeostasis through three distinct ER stress-sensor proteins: inositol-requiring protein-1 (IRE1), and PKR-like ER kinase (PERK), activating transcription factor 6 (ATF6), which are not independent but constitute a complex signaling network (Song et al., 2018). Under physiological conditions, the three sensor proteins are inactivated by binding with the glucose-regulated protein 78 kDa/Bip (GRP78/Bip). As unfolded proteins accumulate in the ER lumen, the chaperone protein GRP78/Bip is released from IRE1α, PERK, and ATF6 and then activates their downstream signaling to relieve the burden of ER (Schroder and Kaufman, 2005). Our previous study has demonstrated that FMDV infection induces ER stress to facilitate virus replication by suppressing the IRE1α pathway (Han et al., 2019). However, the relationship between FMDV infection, ER stress-driven UPR, and the downstream mechanisms are still unclear.

Autophagy, a dynamic intracellular vesicle process, is a double-edged sword that either helps host cell degrade these misfolded/unfolded proteins, damaged organelles, or intracellular pathogens *via* the fusion with lysosomes or is hijacked by invasive viruses to promote their replication (Yorimitsu and Klionsky, 2005; Mizushima, 2007). Although both autophagy and ER stress-driven UPR can function independently from each other during viral replications, increasing researches have shown that all three UPR pathways of IRE1α, PERK, and ATF6 are responsible for the subsequent autophagy induction at different stages of its formation and that the three cellular pathways also participate in the replication and pathogenesis of some viruses *via* the induction of UPR-autophagy pathways. For instance, dengue virus (DEV) and Bluetongue virus (BTV) induce ER stress-mediated autophagy dependent on the two ER stress sensors PERK and IRE1α to facilitate viral replication (Lv et al., 2015; Datan et al., 2016; Yin et al., 2017; Lee et al., 2018). Also, the interaction of FMDV 2C protein with Beclin1 prevented the fusion of lysosomes to autophagosome-containing FMDV, allowing virus replication (Gladue et al., 2012). Meanwhile, an ambiguous study showed that FMDV 3C protease degraded the Atg5-Atg12 complex to suppress autophagy later stage of FMDV replication (Fan et al., 2017). Therefore, the molecular mechanisms involved in FMDV-induced autophagy and the relationship between ER stress-driven UPR, autophagy, and FMDV replication still need further investigations.

Sec62, the transport complex to regulate protein importing into ER, is an ER membrane-associated autophagy receptor with ER-resident LC3-interacting regions (LIR) to promote the delivery of select ER domains to autolysosomes for degradation (Fumagalli et al., 2016). Moreover, we had previously found that Sec62 protein was slightly increased at the early stage of FMDV infection but dramatically decreased later (Guo et al., 2015; Han et al., 2019). Meanwhile, it was also demonstrated that Sec62 positively regulated RIG-1-IFN-β signaling through regulating the stable expression of IRE1-ER stress sensors during FMDV infection (Han et al., 2019). Thus, we speculate that Sec62 might regulate the balance between ER stress and autophagy to affect FMDV replication. In this study, we found that FMDV-induced ER stress triggers autophagic activity through the ATF6 UPR pathway. Moreover, it was also demonstrated that Sec62 could promote phosphorylation activity of IRE1α, then activate the IRE1α-JNK pathway to participate in autophagy and suppress FMDV replication, but FMDV downregulates the expression of Sec62 and IRE1α to achieve its replication. Simultaneously, it was also found that Sec62 colocates and interacts with LC3 to attenuate ER stress and FMDV replication by recruiting and delivering autophagosomes into the lysosome for clearance. Altogether, this study will help us better understand the mechanism of FMDV-host interactions and antiviral therapies against FMDV.

## Materials and Methods

### Cells and Viruses

PK-15 (porcine kidney) was maintained in Dulbecco’s modified Eagle’s medium (DMEM) (Gibco, Carlsbad, CA, USA) supplemented with 10% fetal bovine serum (FBS) (Gibco, CA, USA) and penicillin-streptomycin (100 U/ml and 100 μg/ml, respectively) (Gibco, CA, USA) at 37°C in 5% CO_2_.

FMDV serotype O/BY/CHA/2010 strain (GenBank Accession No. JN998085.1) was preserved by the OIE/National Foot-and-Mouth Disease Reference Laboratory (Lanzhou, China). Viral titers were determined by a 50% tissue culture infective dose (TCID_50_) assay.

### Antibodies and Reagents

Anti-Bip rabbit polyclonal antibody (ab21685), anti-beta actin mouse monoclonal antibody (ab8226), anti-CHOP rabbit monoclonal antibody (ab179823), anti-Sec62 rabbit monoclonal antibody (ab140644), anti-Beclin-1 rabbit monoclonal antibody (ab207612), anti-IRE1 rabbit polyclonal antibody (ab37073), anti-IRE1 (phosphor S724) rabbit monoclonal antibody (ab124945), anti-calnexin rabbit polyclonal antibody (ab22595), and anti-GFP rabbit monoclonal antibody (ab183734) were purchased from Abcam (Cambridge, UK). Anti-Bcl-2 mouse monoclonal antibody (sc-7382), anti-JNK mouse monoclonal antibody (sc-7345), and anti-flag mouse monoclonal antibody (sc-166355) were purchased from Santa Cruz Biotechnology (Santa Cruz, CA, USA). Anti-LC3B rabbit polyclonal antibody (#2775), anti-PERK rabbit monoclonal antibody (#3192), anti-ATF-6 rabbit monoclonal antibody (#65880), anti-ATG5 rabbit monoclonal antibody (#12994), anti-phosho-JNK (Thr183/Tyr185) mouse monoclonal antibody (#9255), anti-LAMP1 mouse monoclonal antibody (#15665), and anti-phosho-Bcl-2 (Ser70) rabbit monoclonal antibody (#2827) were purchased from Cell Signaling Technology (CST, Beverly, MA, USA). Thapsigargin (TG), 4-phenyl butyric acid (4-PBA), 3-methyladenine (3-MA), rapamycin (RAPA), and the secondary antibodies conjugating with HRP, FITC, and TRITC were purchased from Sigma-Aldrich (St. Louis, MO, USA). Alexa Fluor 647-goat anti-mouse IgG (H+L) secondary antibody was purchased from Invitrogen. Polyclonal pig antiserum against FMDV was prepared in our laboratory.

### Plasmid Transfection and RNA Interference

The full-length cDNA encoding XBP1u and XBP1s were amplified from PK-cells and inserted into the pEGFP vector, kindly provided by Dr. M. Zerial (Max Planck Institute, Dresden, Germany). Porcine Flag-IRE1α, Flag-Sec62, and GFP-LC3 were stored in our laboratory. According to the manufacturer’s protocol, these overexpression plasmids and empty vector (EV) were transfected into PK-15 cells using Lipofectamine LTX (Invitrogen, CA, USA). Cells were then infected with 1 MOI of FMDV at 24 h posttransfection, harvested at indicated time points postinfection, and subjected to Western blot analysis, immunofluorescence.

Small interfering RNA (siRNA) targeting PERK, ATF6, Sec62, and negative control (NC) siRNA were synthesized by Genepharma (Shanghai, China). The sequences were used in the siRNAs targeting specific genes were: porcine ATF6 siRNA, 5′-GCAGAACCUCAACCACUUU-3′; porcine PERK siRNA, 5′-GCACU GGUGGAAGGAAAUATT-3′ and 5′-GCAGAUCACUAGUGAUUAU-3’; porcine Sec62 siRNA, 5′-CCAAGUUUCUUCGAUUCAA-3′; NC siRNA, 5′-UUCUCCG AACGUGUCACGU-3′. According to the manufacturer instructions, cells grown to 60% confluent were transfected with related siRNA using Lipofectamine RNA interference (RNAi) MAX (Invitrogen, CA, USA). Cells were incubated for 36 h and exposed to FMDV infection at 1 MOI, then harvested at indicated time points post-infection and subjected to Western blot analysis.

### Western Blotting and Quantification Value Analysis

Protein samples were prepared by adding 100 µl RIPA buffer (Beyotime Biotechnology, Shanghai, China) to each well of a 12-well plate and collected into a new tube. Cell lysates were centrifuged at 12,000 rpm at 4°C for 10 min. The protein concentration was determined by NanoDrop One (Thermo Scientific). Protein samples were boiled at 100°C for 10 min, separated on 10%–12% SDS-PAGE gels, and transferred onto polyvinylidene difluoride (PVDF) membranes (Amersham, Piscataway, NJ, USA). Membranes were blocked in 5% skim milk (Sigma-Aldrich, USA) diluted in TBS containing 0.1% Tween-20 (TBST) at room temperature for 1 h, then incubated for 2 h at room temperature with specific primary antibodies. Next, the membranes were washed three times in TBST, followed by incubation with HRP-conjugated secondary antibody for 1 h. The signals were detected by exposing X-ray film after incubation with ECL Plus reagents (PerkinElmer Life Sciences, Waltham, MA, USA). All Western blotting bands were analyzed using ImageJ software to obtain the corresponding gray value, and the statistical significance of the quantification value was determined by the Student’s *t*-test or ANOVA. Between two and four biological replicates of each blot were performed.

### Indirect Immunofluorescence Assay

PK-15 cells were washed twice with PBS and fixed with 4% paraformaldehyde in PBS for 20 min at room temperature. After fixation, the cells were washed twice and permeabilized with 0.1% TritonX-100 in PBS for 20 min at room temperature. After washing, cells were blocked with PBS containing 5% newborn bovine serum (NBS, Gibco, USA) for 1 h at 37°C and were incubated with the specific primary antibodies at 4°C overnight. The cells were washed with PBS and stained with FITC or TRITC-labeled secondary antibody diluted in PBS with 3% NBS for 1 h at 37°C. Followed by washing six times with PBS, the cells were stained with 2,4-diamidino-2-phenylindole (DAPI) (Molecular Probe, CA, USA) at the concentration of 1 μg/ml for 20 min at room temperature. The cells were then washed three times with PBS, stored in PBS, and examined under a laser-scanning confocal microscope (LSCM, Leica SP8, Solms, Germany) with a Plan-Novofluar ×100/1.4 oil objective. The quantification of puncta was determined by referring to the method in this paper (Lewy et al., 2020).

### RAN Extraction and qRT-PCR Analysis

According to the manufacturer’s protocol, total cellular RNA from PK-15 cells was extracted using TRIzol reagent (Invitrogen), and 1μg of RNA was reverse transcribed with PrimeScript 5× RT Master Mix (TaKaRa, Kusatsu, Japan). qPCR was then performed with SYBR^®^ Premix Ex Taq II Mix (Tli RNaseH Plus) (TaKaRa) by an Applied Biosystems 7500 RealTime PCR system. GAPDH was used as the endogenous reference. The primers specific for the GAPDH were 5′-ACATGGCCTCCAAGG AGTAAGA-3′ (sense) and 5′-GATCGAGTTGGGGCTGTGACT-3′ (antisense). The primers specific for the FMDV were 5′-CAAACCTGTGATGGCTTCGA-3′ (sense) and 5′-CCGGTAC-TCGTCAGGTCCA-3′ (antisense). All samples were run in triplicate, and the experiment was repeated three times.

### TCID_50_

The virus stock samples were serially diluted 10 times with DMEM without FBS and added into 96-well plates with 100′µl/well from 10^−1^ to 10^−7^ in turn. Then, 100′µl PK-15 cell suspensions (density: 1.5′×′10^6^/ml) were added to each well. After incubation at 37°C with 5% CO_2_ for 48′h, the cytopathic effect (CPE) for each well was observed by microscope. Virus titers were calculated according to Reed-Muench and expressed as TCID_50_ per milliliter. MOI was calculated based on TCID_50_.

### Cell Viability Assay

After the PK-15 cells in 96-well plates reached 70% confluence, the plasmid was transfected into cells. Empty vector (EV) was used as control. Furthermore, specific siRNAs were transfected into PK-15 cells. Meanwhile, negative control NC siRNA was used as control. The number of viable cells was estimated at 24 h posttransfection with an MTS assay (CellTiter 96 Aqueous One Solution, Promega, Madison, WI, USA). Subsequently, the absorbance at 490 nm of each well was measured using a microplate reader.

### Coimmunoprecipitation

PK-15 cells were lysed with RIPA lysis buffer containing a protease inhibitor cocktail on ice. Following lysate centrifugation, the supernatant was incubated overnight with anti-GFP antibody and protein G-Sepharose beads (GE Healthcare, 17-0618-01). The Sepharose beads were washed three times with lysis buffer containing 1% NP-40 (Beyotime, P0013F). Co-IP was subsequently analyzed by Western blotting.

### Statistics Analysis

All data were collected in three independent experiments at least and were presented as the mean ± standard deviation (SD) of the results of triplicate experiments. The Student’s *t*-test or ANOVA were used to perform the statistical analyses. Statistical significance was assessed based on the *p*-value: *p < 0.05, **p < 0.01, and ***p < 0.001.

## Results

### FMDV-Induced ER Stress Promotes Autophagy Activation

**Figure 1:**
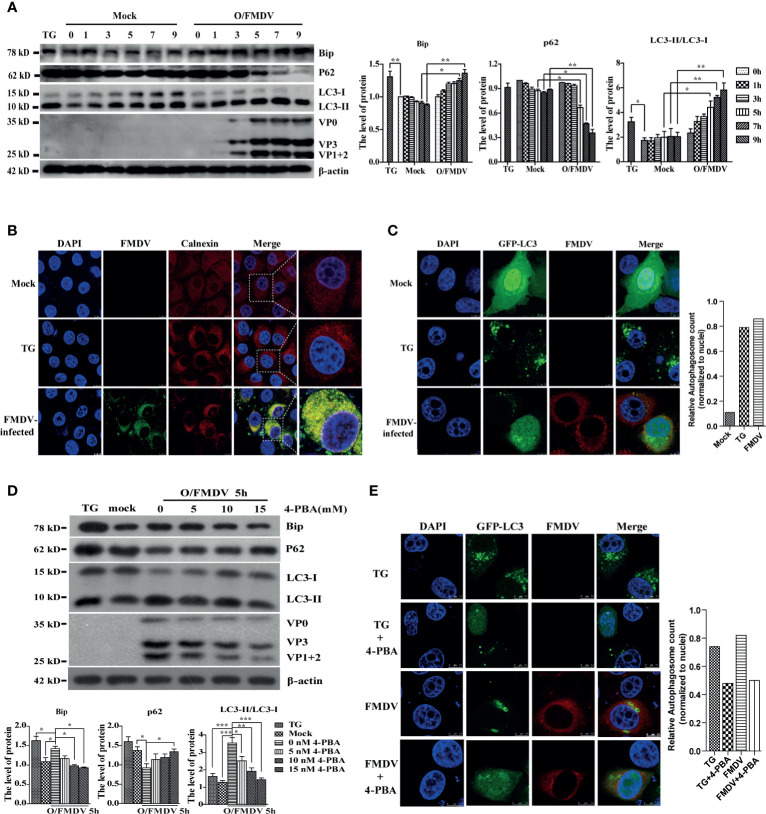
FMDV infection induces ER stress and autophagy activity. **(A)** The Bip, LC3, P62, and FMDV structure proteins were detected by Western blot at indicated time points. The right graph shows the band intensities of specific proteins by densitometry using ImageJ software. The levels of related proteins were normalized to β-actin; data were expressed as means ± S.D of three experiments. Every treatment group compared with control groups, respectively; *p < 0.05, **p < 0.01. **(B)** FMDV-infected and TG-treated cells were fixed at 9 hpi, and mock-infected cells were used as a negative control. Cells were then imaged using confocal microscopy with a ×100 oil-immersion objective lens to detect the aggregation of calnexin. Calnexin was stained with TRITC, FMDV stained with FITC, and nuclei were stained with DAPI. Scale bars: 7.5 μm. **(C)** TG-treated and FMDV-infected PK-15 cells were transfected with GFP-LC3 recombinant plasmid and fixed at 9 hpi for immunofluorescence. The GFP-LC3 puncta aggregation was observed under a laser confocal microscope. FMDV and nuclei were stained with TRITC and DAPI, respectively. Scale bars: 7.5 μm. The puncta quantification was determined by the cells with LC3 fluorescent puncta normalized to the number of nuclei. **(D)** PK-15 cells were pretreated with different concentrations of ER stress inhibitor 4-PBA for 3 h and infected with FMDV for 5 h. Cells were collected for detecting the levels of Bip, LC3, and P62 by Western blot. The levels of related proteins were normalized to β-actin; data were expressed as means ± SD of three experiments. Every treatment group compared with control groups, respectively; *p < 0.05, **p < 0.01, ***p < 0.001. **(E)** the GFP-LC3 puncta aggregation was imaged by laser confocal microscope. FMDV was stained with TRITC and nuclei with DAPI. Scale bars: 7.5 μm. The puncta quantification was determined by the cells with LC3 fluorescent puncta normalized to the number of nuclei.

FMDV is a fast-replicating RNA virus that utilizes the endoplasmic reticulum (ER) to translate many viral proteins rapidly. These excess FMDV proteins induce ER stress and UPR, in turn promoting autophagy to restore cellular homeostasis. To explore the relationship among ER stress, autophagy, and viral replication in FMDV-infection PK-15 cells with 2 MOI, the level of Bip (ER stress marker), LC3-II, and P62 (autophagy marker) were examined by Western blotting at 0, 1, 3, 5, 7, and 9 h postinfection (hpi), respectively. As shown in **Figure 1A**, FMDV infection gradually increased the expression of Bip and LC3-II proteins and decreased P62 levels along with infection time course compared with the mock group. As a positive control for ER stress, PK-15 cells were treated with ER stress inducer Thapsigargin (TG) for 9 h in the parallel experiment. Consistent with the results of FMDV infection, Bip and LC3-II proteins were also increased, and the P62 was reduced in TG-treated cells. These results suggest that FMDV infection induces ER stress and autophagy activity.

Calnexin (ER membrane protein) and GFP-LC3 proteins were visualized by the laser confocal fluorescent microscope in PK-15 cells to verify this phenomenon further. As expected, calnexin was evenly distributed in the cytoplasm in untreated cells, whereas FMDV-infected and TG-treated cells displayed a significant accumulation of fluorescent signals in the perinuclear area. Moreover, almost all of calnexin (red fluorescent puncta) and FMDV protein (green fluorescent puncta) were colocalized in the FMDV-infected cells (**Figure 1B**). GFP-labeled proteins initially have a nuclear to perinuclear location but translocate into the cytoplasm after infection or TG treatment where it appears punctate (**Figure 1C**). These findings also reveal that ER stress and autophagy exactly occur under FMDV infection.

Next, the ER stress inhibitor 4-phenyl butyric acid (4-PBA) was used to explore whether ER stress affects FMDV-induced autophagic activity. PK-15 cells were infected with 1 MOI of FMDV after 4-PBA pretreated at different concentrations (5, 10, and 15 mM) for 3 h. Bip, P62, LC3, and FMDV structural protein expression were examined by Western blotting at 5 hpi. Compared with the TG-treated and FMDV infection cells, Bip levels, the conversion of LC3-I to LC3-II, P62 degradation, and FMDV replication were generally decreased in a dose-dependent manner 4-PBA-treated cells (**Figure 1D**), indicating that 4-PBA inhibits the autophagic formation and FMDV replication. Immunofluorescence was performed to confirm the GFP-LC3 puncta formation by laser confocal microscopy to visualize the inhibitory effect of 4-PBA on autophagic activity during FMDV infection. Our data show that blocking ER stress decreased the GFP-LC3 spots formation in TG-treated and FMDV-infected cells (**Figure 1E**). Altogether, these results reveal that FMDV-induced ER stress triggers autophagic activity and viral replication.

### Autophagy Positively Regulates FMDV Replication

**Figure 2:**
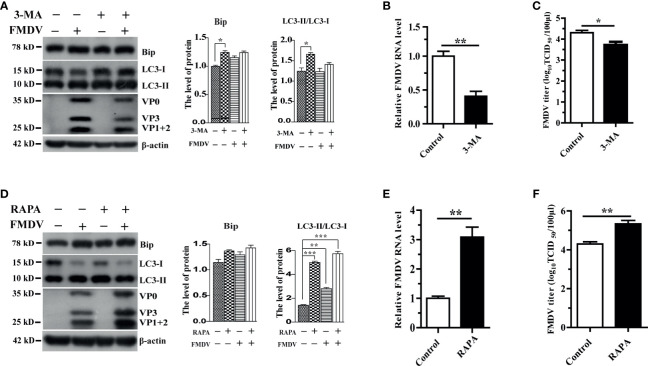
The effect of autophagy on FMDV replication in pharmacologically treated PK-15 cells. **(A)** PK-15 cells were treated with a complete medium containing 3 mM 3-MA for 5 h and then infected by FMDV. Cell samples were processed and subjected to Western blotting at 5 hpi. The existence of 3-MA was indicated with “+.” The levels of related proteins were normalized to β-actin; data were expressed as means ± SD of three experiments. Every treatment group compared with control groups, respectively; *p < 0.05, **p < 0.01. **(B**, **C)** Viral mRNA and viral progeny yields were examined based on qRT-PCR analysis and TCID_50_; **(D)** 500 nM RAPA-treated PK-15 cells were infected by FMDV; and cell lysates were harvested for Western blot analysis at 5 hpi. The existence of RAPA was indicated with “+.” The levels of related proteins were normalized to β-actin; data were expressed as means ± SD of three experiments. Every treatment group compared with control groups, respectively, *p < 0.05, **p < 0.01, ***p < 0.001. **(E**, **F)** Viral mRNA and viral progeny yields were examined based on qRT-PCR analysis and TCID_50_. Data are analyzed as mean ± SD; *p < 0.05, **p < 0.01.

Treatment of PK-15 cells with ER stress inhibitor 4-PBA reduced LC3-II and FMDV structural protein (**Figure 1D**). This observation suggested that autophagy plays a major role in FMDV replication. To substantiate the relationship between autophagy and FMDV replication, the expression levels of Bip, LC3, FMDV structural protein, the viral mRNA, and viral progeny yields were examined after treatment with 3-methyladenine (3-MA), a widely used autophagy inhibitor, and autophagy inducer rapamycin (RAPA), respectively. Results showed that 3-MA treatment reduced the expression of LC3-II and FMDV structural protein and decreased the viral mRNA and viral progeny yields in FMDV-infected PK-15 cells compared with the levels in control cells at 5 hpi (**Figures 2A** **–C**). In contrast, RAPA treatment significantly increased the conversion of LC3-II protein and the expression of FMDV structural protein compared with untreated groups (**Figure 2D**), and RAPA-treated cells caused a significant increase in viral mRNA and FMDV titers (**Figures 2E, F** **)**. Therefore, these results indicate that autophagy positively regulates FMDV replication.

### The ATF6 Pathway of UPR Participates in FMDV-Induced Autophagy

**Figure 3:**
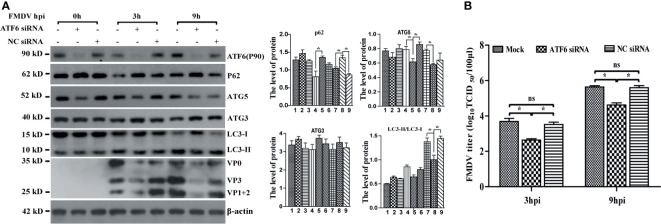
Knockdown of *ATF6* gene inhibits FMDV-induced autophagy and viral replication. **(A)** PK-15 cells were transfected with ATF6-specific siRNA and NC siRNA against endogenous *ATF6* gene, followed by FMDV infection. Total proteins were collected at directed time points and subjected to immunoblotting analysis of ATF6, P62, ATG5, ATG3, LC3, FMDV structure proteins, and β-actin. The levels of related proteins were normalized to β-actin; data were expressed as means ± SD of three experiments. Every treatment group compared with control groups, respectively; *p < 0.05, ns, no significance. **(B)** The viral titers were determined by TCID_50_ in ATF6-knocked down cells.

We have previously reported that FMDV infection-induced ER stress by UPR signaling pathways, in which PERK and ATF6 signaling were activated, whereas the IRE1α pathway was suppressed by decreasing IRE1a level (Han et al., 2019). To confirm whether FMDV-induced autophagy occurs through UPR, PERK- and ATF6-specific siRNAs were transiently transfected into PK-15 cells to knockdown endogenous PERK and ATF6, respectively. As shown in **Supplementary Figure 1**, the expression level of PERK was reduced but did not affect the conversion of LC3-I to LC3-II, the expression of P62 and FMDV structural proteins, as well as viral titer, compared with untransfected cells (mock) and control siRNA-transfected cells (NC siRNA) at every time points in FMDV-infected PK-15 cells (**Supplementary Figures 1A, B**). To verify whether autophagy occurred due to the ATF6 UPR pathway in FMDV-infected PK-15 cells, we next detected the effect of ATF6 knockdown on FMDV-induced autophagy. Compared with mock and NC siRNA-transfected cells, knockdown of ATF6 reduced the conversion of LC3-I to LC3-II in 3 and 9 hpi (no effect in 0 hpi), the structural proteins of FMDV and viral titer were also significantly decreased in the ATF6-knockdown cells (**Figures 3A, B** **)**. The result indicated that the ATF6 pathway of UPR indirectly participates in FMDV-induced autophagy.

Meanwhile, the expression levels of crucial autophagy-related genes P62, ATG3, and ATG5 were also examined in FMDV-infected PK-15 cells. Western blotting analysis showed that the protein level of P62 was increased, while that of ATG5 was decreased in ATF6-depleted cells, but ATG3 was not significantly affected (**Figure 3A**), suggesting that the knockdown of ATF6 reduced autophagosome formation. Collectively, these results revealed the indirect role of the ATF6 UPR pathway in modulating autophagy during FMDV infection, while the PERK branch does not affect autophagy and viral replication.

### FMDV Infection Inhibits the IRE1α-JNK Signaling Pathway

**Figure 4:**
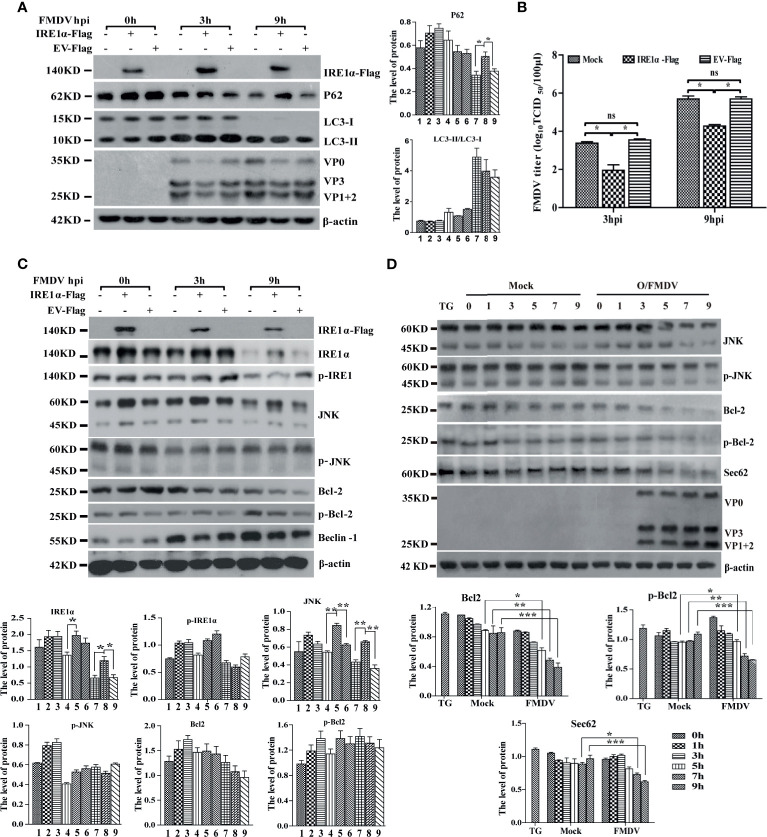
IRE1α-JNK signaling pathway was involved in FMDV-induced autophagy and viral replication. **(A)** PK-15 cells were transfected with cognate Flag-IRE1α and empty vector (EV) for 24 h, followed by FMDV infection. Cell lysates were harvested and subjected to Western blot analysis. The levels of related proteins were normalized to β-actin, data were expressed as means ± SD of three experiments. Every treatment group compared with control groups, respectively; *p < 0.05, **p < 0.01. **(B)** The viral titers were determined by TCID_50_ in IRE1α-overexpressed cells. Data are analyzed as mean ± SD; *p < 0.05, **p < 0.01. **(C)** The effect of IRE1α overexpression on IRE1α-JNK pathway downstream effectors was detected by immunoblotting analysis, containing p-IREα, JNK, p-JNK, Bcl-2, p-Bcl-2 and beclin-1, and β-actin. The levels of related proteins were normalized to β-actin; data were expressed as means ± SD of three experiments. Every treatment group compared with control groups, respectively; *p < 0.05, **p < 0.01. **(D)** The levels of IRE1α-JNK pathway-related proteins and Sec62 were detected by Western blot in FMDV-infected PK-15 cells. The levels of related proteins were normalized to β-actin; data were expressed as means ± SD of three experiments. Every treatment group compared with control groups, respectively; *p < 0.05, **p < 0.01, ***p < 0.001. ns, no significance.

IRE1α is a transmembrane protein localized to ER with ribonuclease and kinase activity. Under ER stress, the accumulation of unfolded proteins induces phosphorylation and oligomerization of IRE1α, which then regulates the splicing of XBP1u mRNA to produce XBP1s and the activation of IRE1α- Jun-N-terminal kinase (JNK) signaling pathways (Jheng et al., 2014). Porcine-driven XBP1u and XBP1s with EGEP tags were constructed to investigate the role of XBP1 in FMDV infection. PK-15 cells were transfected with porcine-driven XBP1u-EGEP or XBP1s-EGEP, respectively, and then infected with FMDV. As shown in **Supplementary Figure 2**, XBP1u and XBP1s were successfully overexpressed in FMDV-infected PK-15 cells. Results indicated that neither of them enhances the development of autophagy and viral replication in FMDV-infected PK-15 cells. We further assess the role of IRE1α-JNK signaling pathways on FMDV infection and construct a porcine-derived IRE1α recombinant plasmid with Flag tag. Our results showed that the overexpression of IRE1α did not affect the conversion of LC3-I to LC3-II but significantly decreases the expression of FMDV proteins and viral titer (**Figures 4A, B** **)**.

Further study indicated that the overexpression of IRE1α increases the expression level of JNK but does not significantly affect the phosphorylation of IRE1α, JNK, and Bcl-2 and the expression of autophagy-related protein Beclin-1 in IRE1α-JNK signaling pathway at different time points (**Figure 4C**), implying that IRE1α-JNK signaling pathway cannot be activated. To better understand the role of IRE1α-JNK signaling pathways in FMDV replication, we further explore the dynamic changes of the IRE1α-JNK signaling pathway in FMDV-infected PK-15 cells. Western blotting analysis indicated that the expression and phosphorylation levels of JNK and Bcl-2 also were gradually reduced with FMDV infection (**Figure 4D**). These results suggest that FMDV infection inhibits the phosphorylation of IRE1α and IRE1α-JNK signaling pathway.

### Sec62 Enhances the Phosphorylation of IRE1α and FMDV-Induced Autophagy

Based on the above two results that the overexpression of IRE1α did not increase the phosphorylation of IRE1α and the conversion of LC3, we speculate that FMDV infection may inhibit a kinase from phosphorylating IRE1α. Sec62, a well-characterized translocation family complex member, is responsible for importing proteins into the ER to increase ER stress and activate UPR (Smith and Wilkinson, 2017). Meanwhile, it also was reported that Sec62 contains a conserved LC3-interacting region (LIR) as an ER-resident autophagy receptor (Fumagalli et al., 2016). Our previous study has reported that Sec62 promotes IRE1α phosphorylation and enhances antiviral immune response (Han et al., 2019). This study also found that the Sec62 expression level was gradually decreased in FMDV-infected PK-15 cells (**Figure 4D**). Given the above information, we hypothesized that Sec62 participated in autophagy regulation by phosphorylating IRE1α and activating the IRE1α-JNK signaling pathway.

**Figure 5:**
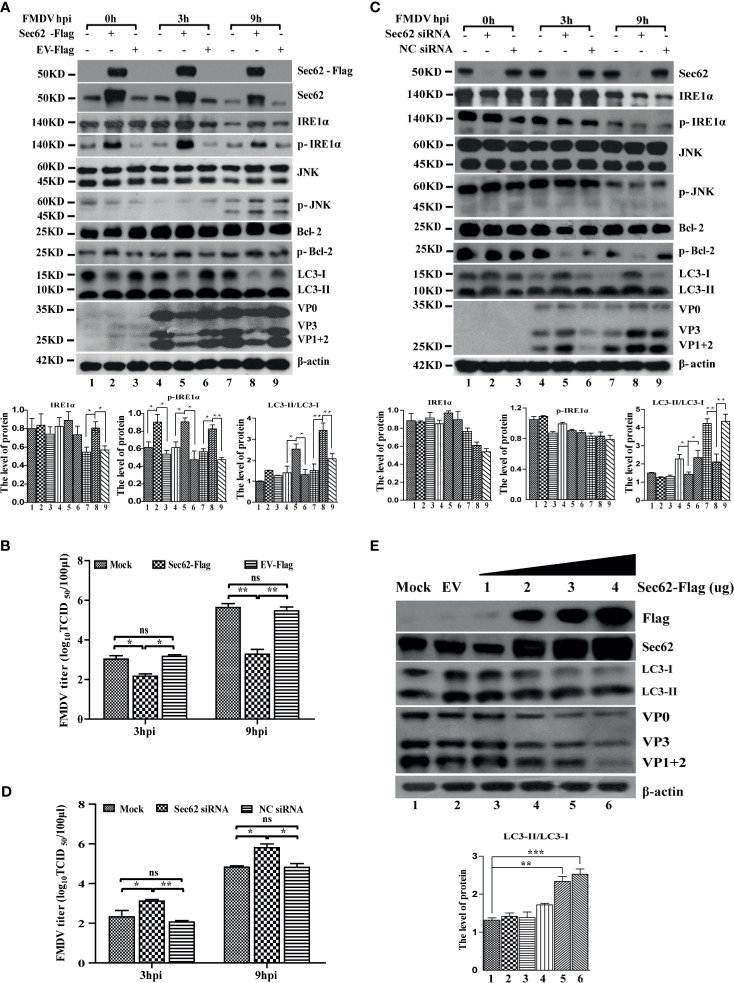
Sec62 promotes autophagy through promoting the phosphorylation of IRE1α. **(A**, **C)** PK-15 cells were transfected with cognate Flag-Sec62 plasmid and EV for 24 h or transfected with NC siRNA and Sec62 siRNA for 36 h, followed by FMDV infection. Cell lysates were harvested at 0, 3, and 9 hpi. The downstream effectors of the IRE1α-JNK pathway signaling were analyzed by Western blotting. The levels of related proteins were normalized to β-actin; data were expressed as means ± SD of three experiments. Every treatment group compared with control groups, respectively, *p < 0.05, **p < 0.01. **(B**, **D)** The viral titers were determined by TCID_50_ in Sec62-overexpressed/knocked down PK-15 cells. Data are analyzed as mean ± SD; *p < 0.05, **p < 0.01. **(E)** PK-15 cells were transfected with the empty vector expressing pCMV-Flag (EV) and increasing Flag-Sec62 expression vector (wedge) concentrations for 24 h. The cell lysates were analyzed by Western blotting. The levels of related proteins were normalized to β-actin; data were expressed as means ± SD of three experiments. Every treatment group compared with control groups, respectively; *p < 0.05, **p < 0.01, ***p < 0.001. ns, no significance.

To investigate the role of Sec62 in FMDV infection, Sec62 was firstly overexpressed or knocked down by transfecting porcine-derived pCMV-Sec62-Flag or specific siRNA targeting Sec62 mRNA into PK-15 cells, respectively. As shown in **Figures 5A, C**, Sec62 was successfully overexpressed and knocked down in FMDV-infected PK-15 cells compared with mock- and EV-/NC siRNA-transfected groups. The overexpression of Sec62 promoted the phosphorylation of IRE1α at late infection times. Furthermore, the phosphorylation level of downstream molecules JNK and Bcl-2 also were consequently increased at 9 h postinfection (**Figure 5A**). Conversely, the phosphorylation levels of IRE1α, JNK, and Bcl-2 were inhibited in Sec62-knocked down cells (**Figure 5C**). Surprisingly, however, FMDV structural proteins and virus titer are significantly decreased, while the conversion of LC3 is increased in Sec62-overexpressed cells (**Figures 5A, B** **)**. Correspondingly, FMDV structural proteins and virus titer are significantly increased, while the conversion of LC3 is decreased in Sec62-knockdown cells (**Figures 5C, D** **)**. Meanwhile, we also do a dose-response experiment of Sec62, and the result shows that Sec62 suppresses FMDV replication but enhances the development of autophagy (**Figure 5E**). Altogether, these results reveal that Sec62 participates in autophagic regulation by activating the IRE1α-JNK signaling pathway and blocks FMDV replication.

### Sec62 Relieves ER Stress by Recruiting and Delivering Autophagosome Into the Lysosome for Clearance

**Figure 6:**
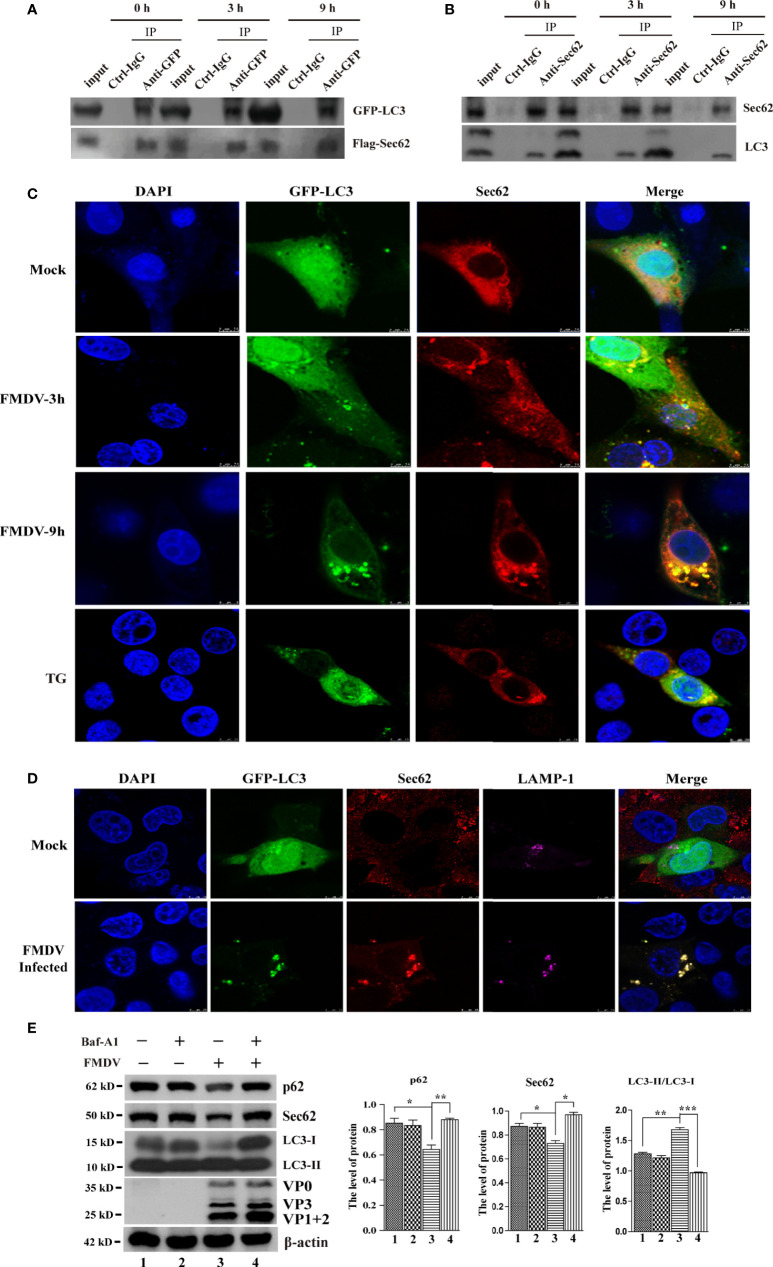
Sec62 interacts with LC3 and recruits autolysosomes. **(A)** PK-15 cells were transfected with cognate Flag-Sec62 and GFP-LC3 for 24 h, followed by FMDV infection at directed time points. Cell lysates were immunoprecipitated with anti-Flag or anti-GFP antibody and control IgG antibody and then analyzed by Western blotting. **(B)** Lysates of FMDV-infected PK-15 cells were prepared, immunoprecipitated with the anti-Sec62 or anti-LC3 antibody and control IgG, and then subjected to Western blot analysis. **(C)** PK-15 cells were transfected with GFP-LC3 for 24 h and then were mock infected or infected with the FMDV virus. Cells were fixed at indicated time points, stained with anti-Sec62 antibody, and then visualized by confocal microscopy. Yellow spots indicate the merging of Sec62 with LC3 puncta. TG-treated cells were included as a positive stimulant. Scale bars: 7.5 μm. **(D)** PK-15 cells were transfected with GFP-LC3 for 24 h and then were mock infected or infected with the FMDV virus. Cells were fixed and subjected to immunostaining with the anti-Sec62, anti-LAMP1, and DAPI, respectively, and then visualized by confocal microscopy. The GFP-LC3 expression signal (green), Sec62 (red), and LAMP1 (pink) were shown. Scale bars: 7.5 μm. **(E)** PK-15 cells were treated with Baf-A1 and then infected by FMDV. Cell samples were processed and subjected to Western blotting at 5 hpi. The existence of Baf-A1 and FMDV was indicated with “+.” The levels of related proteins were normalized to β-actin; data were expressed as means ± SD of three experiments. Every treatment group compared with control groups, respectively; *p < 0.05, **p < 0.01, ***p < 0.001.

A previous study has reported that Sec62 plays a key role in maintaining and recovering ER homeostasis (Fumagalli et al., 2016). Our results also indicate that Sec62 is involved in autophagy in FMDV-infected PK-15 cells. Therefore, we next asked how Sec62 regulates the development of autophagy and whether Sec62 restores ER homeostasis through autophagy. The coimmunoprecipitation assay was firstly performed to explore whether the LIR of Sec62 interacts directly with LC3. PK-15 cells were transfected with the Flag-Sec62 and GFP-LC3, followed by FMDV infection. Cells lysate was harvested at directed time points and carried out immunoprecipitation with anti-GFP antibody. As expected, our results showed that Flag-Sec62 physically interacted with GFP-LC3 (**Figure 6A**).

**Figure 7:**
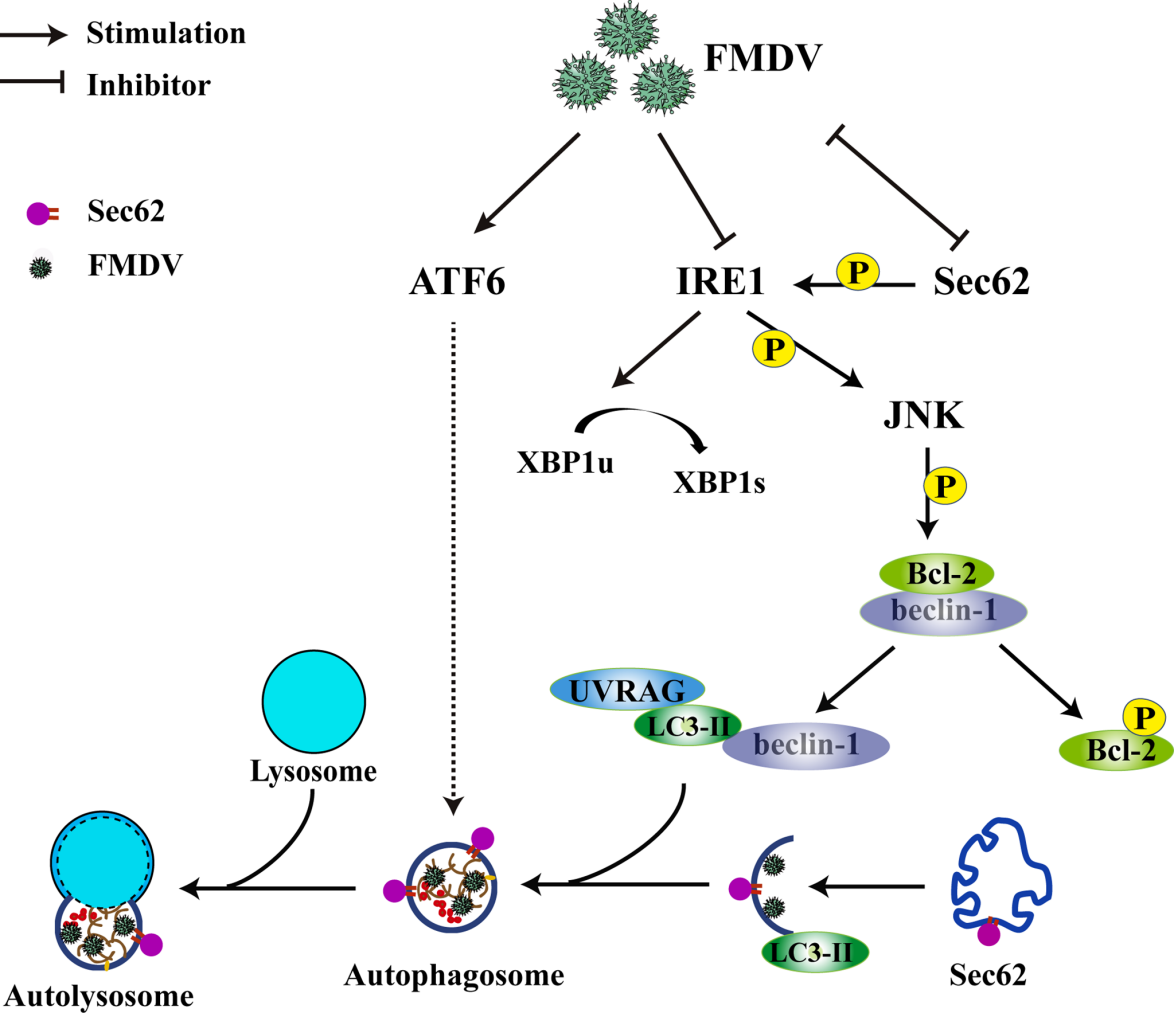
Schematic of how Sec62 regulates FMDV-induced autophagy. FMDV infection activates ATF6 signaling and inhibits the expression of Sec62 and IRE1α. The ATF6 pathway of UPR indirectly participates in FMDV-induced autophagy. Sec62 activates the IRE1α-JNK signaling pathway through phosphorylating IRE1α and recruits autolysosomes to attenuate ER stress and block FMDV replication.

Meanwhile, an anti-Sec62 antibody was used for IP assay to explore the interaction between endogenous Sec62 and LC3. As shown in **Figure 6B**, Sec62 was specially coprecipitated with LC3 at 0, 3, and 9 hpi. The localization of Sec62-Flag with GFP-LC3 puncta was analyzed by immunofluorescence analysis in FMDV-infected PK-15 cells. As shown in **Figure 6C**, Sec62-Flag and GFP-LC3 puncta were evenly distributed in mock-infected cells, whereas FMDV infection results in partial translocation of LC3 from the nucleus to the cytoplasm to form large fluorescence puncta with Sec62 in FMDV-infected cells. Meanwhile, we also found that Sce62 colocalizes with LC3 and the endogenous autolysosomal marker protein LAMP1 (**Figure 6D**). To confirm that Sec62 was also degraded through the autolysosomal systems, PK-15 cells were treated with bafilomycin A1 (BafA1), a specific inhibitor of autophagosome and lysosome fusion. Results showed that BafA1 treatment inhibited Sec62 and p62 degradation, and LC3 conversion, but enhanced FMDV infection, suggesting that Sec62 plays a key role in restoring ER homeostasis by delivering ER components to autolysosomal systems for clearance (**Figure 6E**). Altogether, these results indicate that Sec62 is possibly beneficial for promoting FMDV-induced autophagy through phosphorylating IRE1α and recruiting autolysosomes to attenuate ER stress and block FMDV replication (**Figure 7**).

## Discussion

ER is an elaborate cellular organelle that plays a key role in protein translation, folding, modification, and transportation. In recent years, increasing researches have demonstrated that virus replications lead to an imbalance of ER homeostasis due to the accumulation of misfolded or unfolded proteins (Jheng et al., 2014; Yin et al., 2017; Song et al., 2018). In response to the imbalance, the virus-infected cells trigger the ER stress response and induce the activation of an adaptive UPR to relieve the load of newly synthesized proteins or activate innate antiviral immune response by inducing IFN signaling (Jheng et al., 2014; Yang et al., 2016). Meanwhile, some studies have also reported that virus-induced autophagy is closely related to the three ER stress-associated UPR signaling pathways, such as DEV, CVFV, and JEV (Sharma et al., 2017; Lee et al., 2018; Zhu et al., 2019). In this study, we explored the effect of FMDV infection on ER stress-associated UPRs and autophagy. Our results showed that FMDV-induced ER stress enhances autophagy activity (**Figure 1**) and autophagy plays a positive role in regulating FMDV replication (**Figure 2**). Moreover, we have previously reported that during FMDV infection, among the three ER stress-associated UPR, PERK and ATF6 signaling pathways were activated, while IRE1α-JNK was suppressed. Herein, our results show that the activated PERK-eIF2a pathway does not affect autophagy and FMDV replication (**Supplementary Figure 1**). However, this result contradicts the recently published article (Ranjitha et al., 2020), as different virus strains and cell types may cause this contradictory result. Meanwhile, we also found that the ATF6 pathway participates in FMDV-induced autophagy and viral replication (**Figure 3**), while the IRE1α pathway only affects viral replication (**Figure 4A**).

IRE1α-ER stress sensor is an ER-localized transmembrane protein containing cytosolic kinase and RNase domain. During ER stress, the activated IRE1α forms a complex with tumor necrosis factor receptor-associated factor-2 (TRAF2) and apoptosis signal-regulating kinase-1 (ASK1), which phosphorylates Jun-N-terminal kinase (JNK) (Ron and Hubbard, 2008; Gardner and Walter, 2011) or facilitate X-box binding protein-1 (XBP1u) into a transcriptional activator XBP1s to trigger autophagy through transcriptional activation of Beclin-1 (Margariti et al., 2013). The JNK-mediated phosphorylation of Bcl-2 causes Beclin-1 releasing from Beclin-1/Bcl-2 complex to form the vacuolar protein sorting 34 (Vps34)-Beclin-1 complex, which drives the nucleation of the isolation membrane (Pattingre et al., 2005). However, several studies have reported that the levels of IRE1α and phosphor-IRE1α were gradually decreased during FMDV replication and that the splicing event of XBP1 mRNA was not apparent in FMDV-infected cells, suggesting that the IRE1α/XBP1 pathway was not involved in autophagy and FMDV proliferation (Han et al., 2019; Ranjitha et al., 2020). Consistent with this result, the overexpression of XBP1u and XBP1s do not affect the development of autophagy and viral replication (**Supplementary Figure 2**). Furthermore, IRE1α-dependent RNA decay activates RIG-1 signaling to induce IFN-β antiviral responses (Lencer et al., 2015), therefore, suggesting that the decrease of IRE1α and phosphor-IRE1α level might be a key immune evasion mechanism during FMDV replication. Indeed, we found that the overexpression of IRE1α significantly decreases FMDV replication but does not affect the development of autophagy (**Figure 4A**). In addition to IRE1α/XBP1 and IRE1α-RIDD-RIG-I pathway, IRE1α can also activate the downstream signaling of stress kinases JNK to promote autophagy (Song et al., 2018). However, our results indicated that the overexpression of IRE1α only enhances the expression level of JNK but cannot induce the phosphorylation of IRE1α and JNK by activating the IRE1α-JNK signaling pathway (**Figure 4A, B** **)**. Therefore, we speculate that the activation of the IRE1α-JNK pathway requires a kinase, which is inhibited by FMDV infection.

Sec62, an autophagy receptor, delivers ER components selectively to the autolysosome system for degradation (Birgisdottir et al., 2013; Fumagalli et al., 2016), but it was greatly decreased at the late stage of FMDV infection. Furthermore, our previous study found that the level of IRE1a was stabilized by overexpression of Sec62 in FMDV-infected PK-15 and BHK-21 cells (Han et al., 2019). Thus, it is believed that Sec62 plays a major function in the IRE1α-JNK pathway. When Sec62 was overexpressed, FMDV structural proteins and virus titer were significantly decreased in FMDV-infected PK-15 cells, while the conversion of LC3-II was enhanced. On the contrary, FMDV replication was increased, and the conversion of LC3-II was reduced in Sec62-knocked downed cells (**Figure 5**), indicating that Sec62 negatively regulates FMDV proliferation by activating the IRE1α-JNK signaling pathway and participates in the formation of autophagosomes (**Figure 5**). Moreover, as an autophagy receptor, Sec62 contains a conserved LC3-interacting region. Therefore, we reveal that Sec62 may be a scaffold protein linking ER stress and cellular autophagy to block FMDV replication. Indeed, we did find that Sec62 can colocalize with LC3 by coimmunoprecipitation and immunofluorescence. These results, thus, suggest that Sec62 may be involved in autophagosome degradation through the lysosomal system. As expected, Sce62 colocalizes with LC3 and LAMP1 by immunofluorescence assay (**Figure 6**). Surprisingly, we found that autophagy positively regulates FMDV replication after treatment with 3-MA or RAPA (**Figure 2**), but autophagy has contrasting effects in Sec62-overexpressed or Sec62-knocked down cells on FMDV production. The result further shows that Sec62 promotes the formation of complete autophagic flux, while FMDV infection destroyed the autophagic flux. Although we have demonstrated that Sec62 plays an important role in ER stress, autophagy, and FMDV replication (**Figure 6**), the detailed mechanism of Sec62 in ER stress and autophagy still needs to be further explored.

In conclusion, we report in this study that a novel mechanism of FMDV-mediated autophagy is triggered by the two ER stress-associated UPR of ATF6 and IRE1α-JNK signaling pathways. In addition, we also find that Sec62 can inhibit FMDV proliferation by recruiting autophagosomes to relieve ER stress. In return, FMDV infection attenuates Sec62 expression and autophagosome recruitment (**Figure 7**). Therefore, we deem that Sec62 might be a potential target of antiviral therapy.

## Data Availability Statement

The original contributions presented in the study are included in the article/**Supplementary Material**. Further inquiries can be directed to the corresponding author.

## Author Contributions

HG designed the research. JW and ZZ wrote the paper. ZZ and JW, ZT, SS, and SW performed the experiments. All authors contributed to the article and approved the submitted version.

## Funding

This work was financially supported by the National Natural Science Foundation of China (32072847, 32072859, 32002272, and 31873023) and the Central Public-interest Scientific Institution Basal Research fund (1610312020009 and 1610312020019).

## Conflict of Interest

The authors declare that the research was conducted in the absence of any commercial or financial relationships that could be construed as a potential conflict of interest.

## Publisher’s Note

All claims expressed in this article are solely those of the authors and do not necessarily represent those of their affiliated organizations, or those of the publisher, the editors and the reviewers. Any product that may be evaluated in this article, or claim that may be made by its manufacturer, is not guaranteed or endorsed by the publisher.

## Supplementary Material

Supplementary Figure 1 The effect of *PERK* gene on FMDV-induced autophagy and viral replication in PREK-knockdown cells. **(A)** PK-15 cells were transfected with PERK-specific siRNA and NC siRNA against endogenous *PERK* gene, followed by FMDV infection. Total proteins were collected at directed time points and subjected to immunoblotting analysis of P62, LC3, FMDV structure proteins, and β-actin. **(B)** The viral titers were determined by TCID_50_ in ATF6-knocked down cells. Data are analyzed as mean ± SD; **p* < 0.05, ** *p* < 0.01.

Supplementary Figure 2 XBP1 was not involved in the development of autophagy and FMDV proliferation. **(A**, **C)** PK-15 cells were transfected with XBP1u-EGFP and XBP1s-EGFP for 24 h and followed by FMDV infection. Autophagy-associated proteins were analyzed by Western blotting in FMDV-infected PK-15 cells. **(B**, **D)** The viral titers were determined by TCID_50_ in XBP1u/XBP1-overexpressed PK-15 cells. Data are analyzed as mean ± SD; **p* < 0.05, ***p* < 0.01.
