# Supplementary figures and images for: Sec62 Regulates Endoplasmic Reticulum Stress and Autophagy Balance to Affect Foot-and-Mouth Disease Virus Replication
Source: Front Cell Infect Microbiol. 2021 Aug 31;11:707107. doi: 10.3389/fcimb.2021.707107 (PMC8438241; doi:10.3389/fcimb.2021.707107)

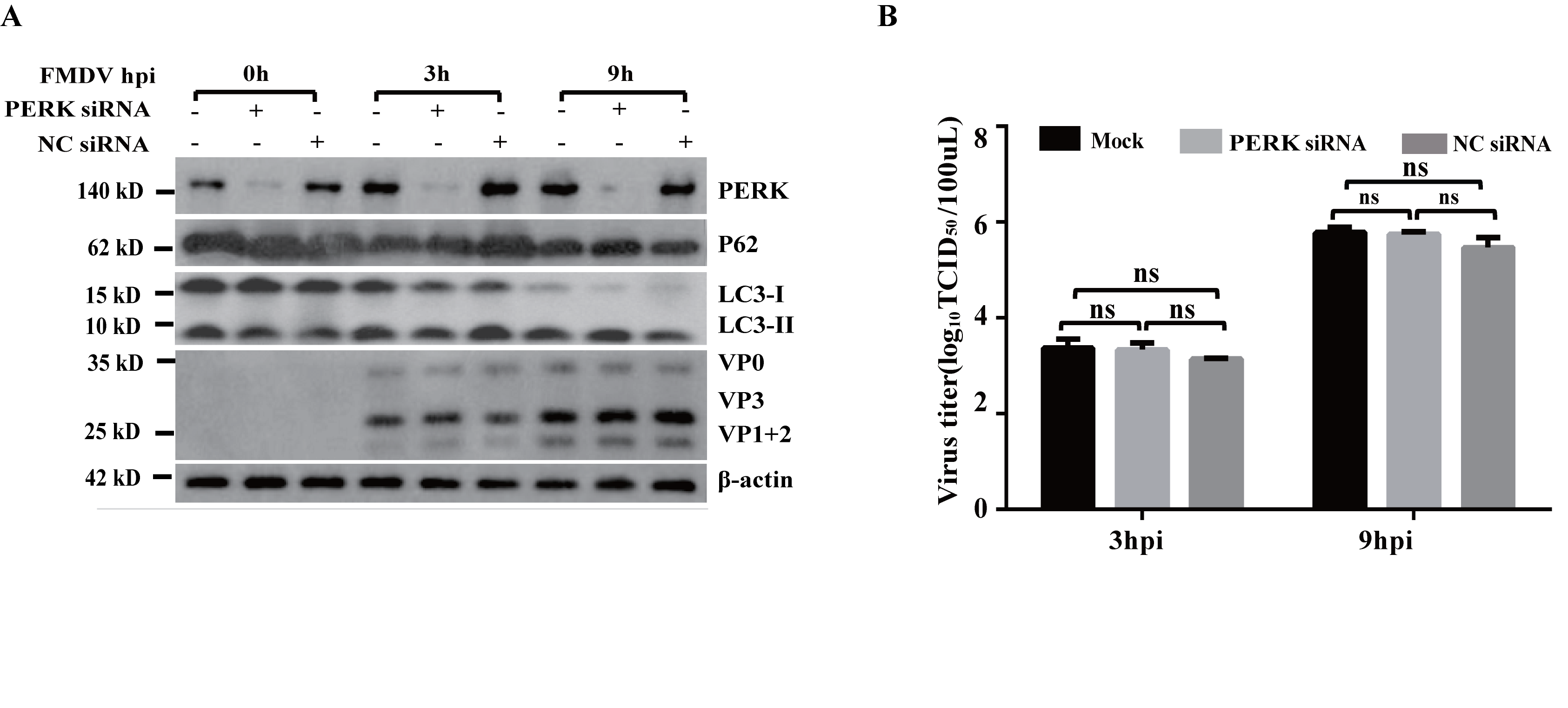

Supplement: Supplementary Figure 1 — The effect of PERK gene on FMDV-induced autophagy and viral replication in PREK-knockdown cells. (A) PK-15 cells were transfected with PERK-specific siRNA and NC siRNA against endogenous PERK gene, followed by FMDV infection. Total proteins were collected at directed time points and subjected to immunoblotting analysis of P62, LC3, FMDV structure proteins, and β-actin. (B) The viral titers were determined by TCID50 in ATF6-knocked down cells. Data are analyzed as mean ± SD; *p < 0.05, ** p < 0.01. [file Image_1.tif]

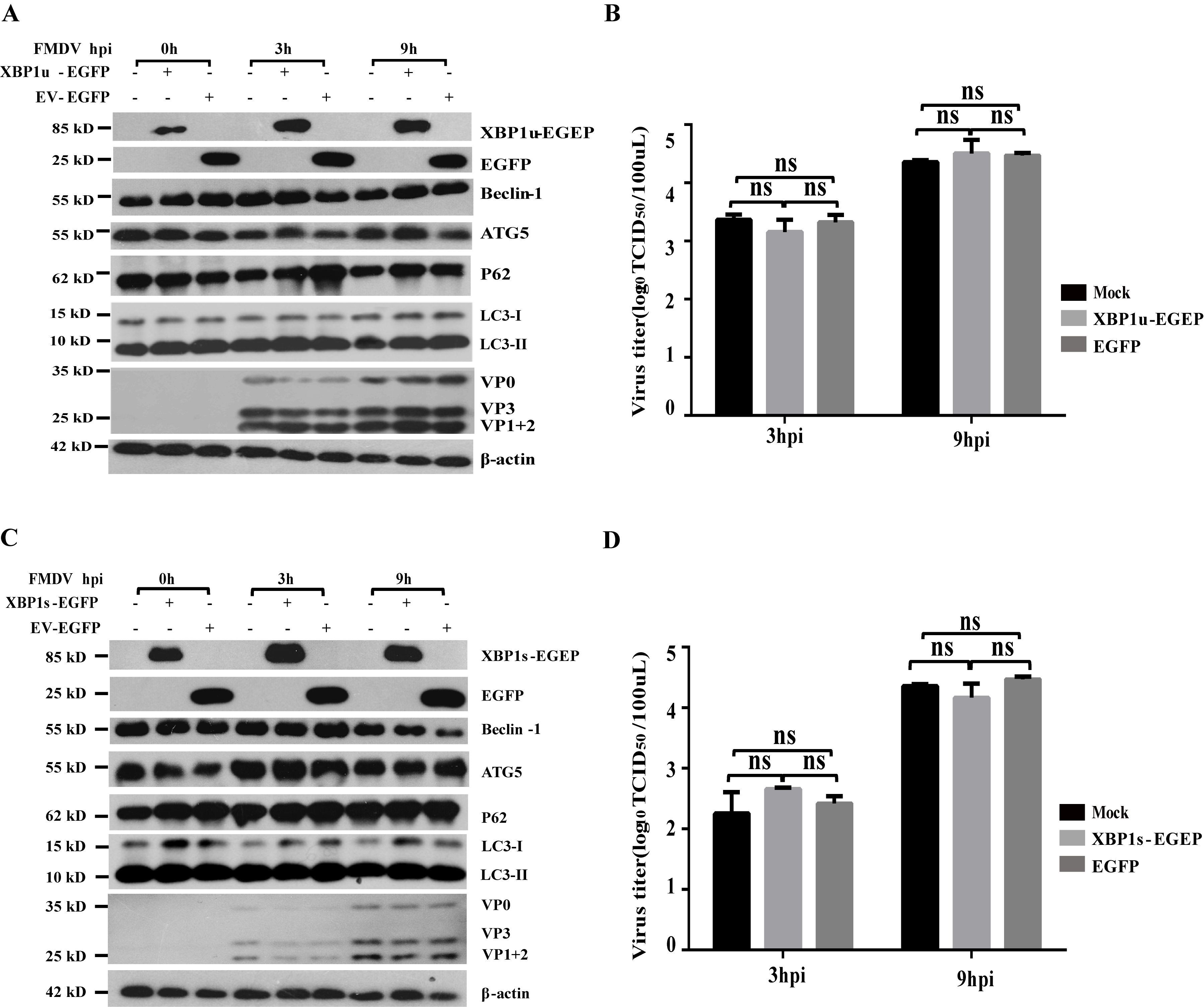

Supplement: Supplementary Figure 2 — XBP1 was not involved in the development of autophagy and FMDV proliferation. (A, C) PK-15 cells were transfected with XBP1u-EGFP and XBP1s-EGFP for 24 h and followed by FMDV infection. Autophagy-associated proteins were analyzed by Western blotting in FMDV-infected PK-15 cells. (B, D) The viral titers were determined by TCID50 in XBP1u/XBP1-overexpressed PK-15 cells. Data are analyzed as mean ± SD; *p < 0.05, **p < 0.01. [file Image_2.tif]
